# Radiomics of US texture features in differential diagnosis between triple-negative breast cancer and fibroadenoma

**DOI:** 10.1038/s41598-018-31906-4

**Published:** 2018-09-10

**Authors:** Si Eun Lee, Kyunghwa Han, Jin Young Kwak, Eunjung Lee, Eun-Kyung Kim

**Affiliations:** 1 Department of Radiology, Severance Hospital, Research Institute of Radiological Science and Center for Clinical Image Data Science, Yonsei University College of Medicine, Seoul, Korea; 2 Department of Computational Science and Engineering, Yonsei University, Seoul, Korea

## Abstract

Triple-negative breast cancer (TNBC) is sometimes mistaken for fibroadenoma due to its tendency to show benign morphology on breast ultrasound (US) albeit its aggressive nature. This study aims to develop a radiomics score based on US texture analysis for differential diagnosis between TNBC and fibroadenoma, and to evaluate its diagnostic performance compared with pathologic results. We retrospectively included 715 pathology-proven fibroadenomas and 186 pathology-proven TNBCs which were examined by three different US machines. We developed the radiomics score by using penalized logistic regression with a least absolute shrinkage and selection operator (LASSO) analysis from 730 extracted features consisting of 14 intensity-based features, 132 textural features and 584 wavelet-based features. The constructed radiomics score showed significant difference between fibroadenoma and TNBC for all three US machines (*p* < 0.001). Although the radiomics score showed dependency on the type of US machine, we developed more elaborate radiomics score for a subgroup in which US examinations were performed with iU22. This subsequent radiomics score also showed good diagnostic performance, even for BI-RADS category 3 or 4a lesions (AUC 0.782) which were presumed as probably benign or low suspicious of malignancy by radiologists. It was expected to assist radiologist’s diagnosis and reduce the number of invasive biopsies, although US standardization should be overcome before clinical application.

## Introduction

Breast cancer consists of heterogeneous subtypes that have distinct morphologic, genetic and clinical characteristics. Triple-negative breast cancer (TNBC) is one subtype that does not express the estrogen receptor (ER) and progesterone receptor (PR) or overexpress the human epidermal growth factor-2 (HER-2). TNBC accounts for 10–27% of whole breast cancers, and presents the highest rate of recurrence and the poorest outcomes^1–5^.

Breast ultrasonography (US) is an important and reliable modality used to diagnose breast cancer. In general, US signs that have a high positive predictive value (PPV) for malignancy according to the American College of Radiology Breast Imaging Reporting and Data System (BI-RADS) lexicon are irregular shape, spiculated/angular margins, marked hypoechogenicity, posterior acoustic shadowing, and a nonparallel orientation^6–8^. However, TNBC tends to have oval or round shapes and circumscribed margins, reflecting a rapidly proliferating tumor with pushing borders prior to significant stromal reaction. It is also more likely to present with posterior acoustic enhancement since highly cellular circumscribed carcinomas tend to have enhanced through-transmission^9–13^. This benign-looking appearance of TNBC might decrease the diagnostic performance of US and delay proper treatment.

Computer-aided diagnosis (CAD) systems have been used as second readers for analyzing breast lesions by using computational algorithms to make more conclusive diagnoses^14–16^. In US images, texture patterns have been regarded as useful features for differentiating malignant and benign tumors^17–19^. Several previous studies were capable of differential diagnosis between benign and malignant breast lesions, using texture features based on US images such as contrast from gray-level co-occurrence matrices (GLCM), correlation from Haralick’s texture features or combination of contrast and covariance from GLCM^17–19^. Radiomics is a natural extension of the texture analysis in that radiomics analyses usually extract hundreds of features from a medical image such as US, CT or MRI. It assumes that extracted imaging data are the product of mechanisms occurring at a genetic and molecular level, possibly connected with tumor behavior or patient’s prognosis^20,21^. It extracts a large number of quantitative features from digital images and unearths the data for hypothesis generation, testing or both. It finds intralesional heterogeneities from imaging data which are indistinguishable to the naked eye^2^ ^22^. Theoretically, US images possibly contain hidden information that can be hardly perceived by radiologists. We hypothesized it might provide guidance for differential diagnosis between benign-looking TNBC and fibroadenoma. Among hundreds of features, a radiomics score is derived from the selected features which reflect or correlate with pathology more effectively using LASSO (least absolute shrinkage and selection operator) regression analysis method.

We evaluated diagnostic performance of developed radiomics score for differential diagnosis between TNBC and fibroadenoma, compared with proven pathology. We expected that it would reduce the number of false-negative US examinations or the number of invasive biopsies eventually.

## Materials and Methods

This retrospective study was approved by the institutional review board (IRB) of Severance Hospital (Seoul, Korea), with a waiver for informed consent.

### Patients and Data acqusition

**Figure 1:**
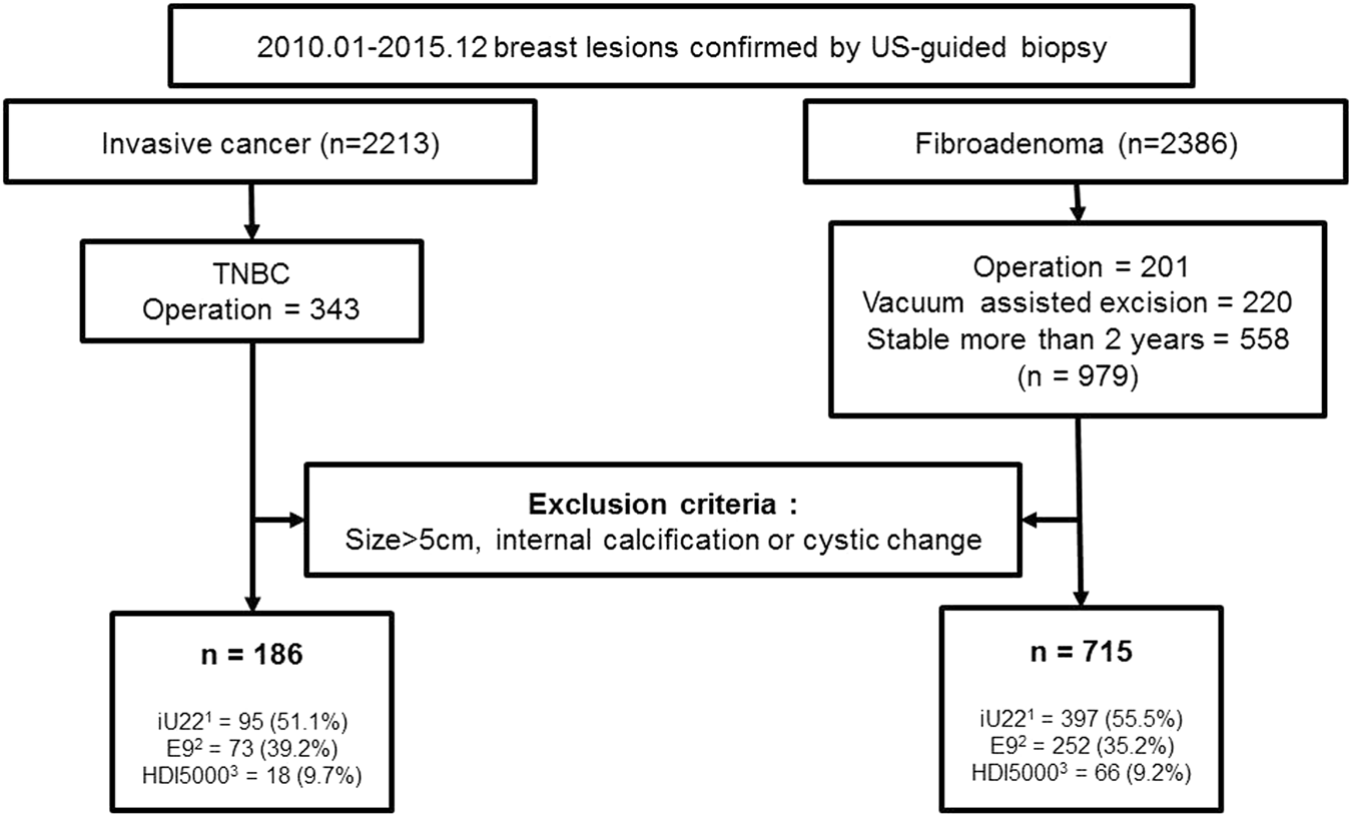
Flowchart for lesion selection. ^1^IU22, Philips Medical Systems, Bothell, WA. ^2^LOGIQ E9, GE Healthcare, Milwaukee, WI. ^3^HDI 5000, Philips Medical Systems, Bothell, WA.

Between January 2010 and December 2015, there were 2213 patients diagnosed with invasive breast cancer after US-guided core biopsy. 343 tumors were finally diagnosed as TNBC based on surgical pathology, which did not express ER and PR or overexpress HER-2^1^. There were 2386 patients diagnosed with fibroadenoma after US-guided core biopsy during the same period. Of them, 979 had tumors definitely confirmed as fibroadenoma; 201 underwent surgical excision, 220 underwent vacuum-assisted excision and 558 had tumors which showed a stable appearance for more than 2 years (Fig. 1).

US images were collected using three different ultrasound machines (iU22 and HDI 5000; Philips Medical Systems, Bothell, WA, LOQIG E9; GE Healthcare, Milwaukee, WI)) with linear array transducers. All US images were preprocessed by a routine setting including log-compression, compound imaging and scan conversion regardless of the type of devices. We excluded the images which were scanned by harmonic imaging. The aim of our study was to supply additional information to aid radiologists in distinguishing fibroadenoma and TNBC based on texture analysis. Therefore, we excluded lesions with calcifications or cystic changes that might have an extreme effect on pixel values. We also excluded tumors larger than 5 cm because these tumors were not fully included in a single plane which made it difficult to define a region-of-interest (ROI).

Finally, we included 186 TNBCs and 715 fibroadenomas from 840 patients (mean age, 42 yr; range, 13–90 yr). The mean lesion diameter was 15 mm (range, 3–49 mm) on US. Among 901 breast lesions, 492 (54.6%) lesions were examined by iU22 and 325 (36.1%) lesions were examined by LOGIQ E9. The rest (84, 9.3%) were examined by HDI 5000. Two radiologists assessed each breast mass according to the US BI-RADS lexicon, and categorized lesions as 3, 4, and 5 by consensus with probability of malignancy risk being less than 2%, 2–95%, and more than 95%, respectively. Category 4 was subdivided into 4a, 4b and 4c with probability of malignancy risk being 2–10%, 10–50% and 50–95%, respectively^8^.

### Feature extraction

A radiologist (S.E.L, a third-year resident) who was blinded to the pathologic results selected one axial image among the ultrasound images of each breast mass and drew a ROI along the mass margin using the Microsoft Paint program.

For the multi-feature-based radiomics study, we extracted a large number of radiomics features including intensity-based features, textural features and wavelet-based features, many of which currently have no known clinical significance. We followed the feature set which was suggested by the previous review article^22^. Intensity-based features describe the distribution of pixel intensities within an US image such as energy, entropy and kurtosis. Texture features were extracted using the gray-level co-occurrence matrices(GLCM) and gray-level run-length matrices (GLRLM) in 0°, 45°, 90°, and 135° which are tabulations of how often different combinations of pixel brightness values (gray levels) occur in an image for each direction respectively^23^. Wavelet transformation decouples textural information by decomposing the original image in low and high frequencies. In this study, using two-dimensional coiflet wavelets, the original US image was decomposed into four decompositions (LH, HH, HL, LL). Finally we attained 730 features consisting of 14 intensity-based features, 132 textural features, 584 wavelet-based features quantifying tumor imaging features by using in-house algorithms implemented by Matlab software (version R2017b, MathWorks).

### Interobserver agreement for defining the region-of-interest (ROI)

ROIs were manually drawn and not found with the segmentation process. To exclude the possibility of interobserver variability affecting the ROI, another staff radiologist (E.K.K, specializing in breast imaging for 22 years) drew ROIs in 50 randomly selected lesions to analyze interobserver reproducibility. Interobserver agreement was evaluated by the intraclass correlation coefficient (ICC) with the 95% confidence interval (CI) based on a two-way random effect model between the 730 feature parameters of the 50 randomly selected lesions.

### Logistic LASSO Regression

Patients were divided into the training and validation set using random sampling and there were no significant differences in the average values of age, lesion size, distribution of BI-RADS category and pathology ratio. Because the number of patients has to be superior to the number of covariates by at least 10 times for multivariate analysis^24^, radiomics features were generated by using the penalized logistic regression with a least absolute shrinkage and selection operator (LASSO) in the training set. The pathology of the breast masses was entered as a dependent variable, Y, in the logistic regression model and the 730 radiomics features were entered as covariates. A tuning parameter (lambda) was selected to maximize the AUC based on 10-fold cross-validation in the training set. A radiomics score was computed by a linear combination of the selected features weighted by each coefficient. Using the formula for the radiomics score defined in the training set, we obtained and compared radiomics scores according to the type of US machine and lesion pathology. Because imaging features based on texture analysis might depend on the type of US machine used, we repeated the LASSO analysis in patients who underwent US examinations with iU22 which was the most commonly used machine. Since breast lesions of BI-RADS category 4b, 4c or 5 are morphologically distinct enough for malignancy to be suspected with the naked eye, we additionally evaluated the performance of the radiomics score in lesions previously diagnosed as BI-RADS category 3 and 4a. Radiomics scores were converted to the predicted probabilities of malignancy (%) for convenient reference. The conversion equation was as follows.

$$\hat{p}=\frac{\exp (0.6985+1.6381\ast radiomics\,score)}{1+\exp (0.6985+1.6381\ast radiomics\,score)}\times 100$$

Statistical analyses were performed using R software, version 3.3.2 (http://www.R-project.org). All tests were two-sided, and p values of less than 0.05 were considered to indicate statistical significance.

The datasets generated and/or analysed during the current study are available from the corresponding author on reasonable request.

## Results

### Clinical characteristics

There were no significant differences between the training and validation set for lesion size, final pathology ratio, or US BI-RADS category (*p* = 0.183–0.964; Table 1). We used Student’s t-test and Chi-square test for comparsion.

**Table 1:** Demographic and Clinical Characteristics of Patients in the Training Data Set and Validation Data Set.

Characteristics	Total			iU 22 subgroup		
	Training set	Validation set	P*	Training set	Validation set	P*
Total number of lesions	454	447		369	123	
Lesion size (average, mm)	15.7	15.2	0.353	15.9	14.7	0.183
Malignancy rate	20.7% (94/454)	20.6% (92/447)	0.964	18.4% (68/369)	22.0% (27/123)	0.391
BI-RADS			0.334			0.580
3	142 (31.3%)	126 (28.2%)		103 (27.9%)	38 (30.9%)	
4a	232 (51.1%)	257 (57.5%)		210 (56.9%)	64 (52.0%)	
4b	30 (6.6%)	20 (4.5%)		18 (4.9%)	10 (8.1%)	
4c	21 (4.6%)	18 (4.0%)		17 (4.6%)	6 (4.9%)	
5	29 (6.4%)	62 (5.8%)		21 (5.7%)	5 (4.1%)	

P* value compared between the training and validation set, Student’s t-test and Chi-square test.

### Interobserver agreement

The interobserver reproducibility of texture feature extraction between the two radiologists for 50 randomly selected lesions was high (ICC, 0.691–1.000; Table 2). Therefore, all outcomes were based on the measurements of the first radiologist.

**Table 2:** Minimum and maximum value of the intraclass correlation coefficient for 730 radiomics features (DWT discrete wavelet transformation, H high, L low).

Degree	Without DWT	DWT_HH	DWT_HL	DWT_LH	DWT_LL
	Intraclass correlation coefficient (Min, Max)				
0	0.864, 1.000	0.808, 1.000	0.903, 1.000	0.985, 1.000	0.912, 1.000
45	0.827, 1.000	0.691, 1.000	0.757, 1.000	0.956, 1.000	0.890, 0.999
90	0.797, 1.000	0.822, 1.000	0.847, 1.000	0.834, 1.000	0.934, 1.000
135	0.886, 1.000	0.890, 1.000	0.891, 1.000	0.941, 1.000	0.907, 1.000

### Construction and validation of the radiomics score

Twenty-three radiomics features were selected to maximize AUC values among a total of 730 features in the training set using the LASSO analysis (Supplementary Table S1). Among the 23 features, the feature which suggested TNBC most strongly was HH_sre_45_90 (high/high, short run emphasis, 90 degree) and the feature which favored fibroadenoma most strongly was LH_imc1_35_90 (low/high, informational measure of correlation 1, 90 degree). The defined radiomics score showed significant difference between fibroadenoma and TNBC for all three US machines (*p* < 0.001, Table 3). This difference was also confirmed in the validation set. However, we found the total median values of the radiomics score varied according to the type of US machine, even though the pathologic ratio did not significantly differ according to the US machine used. Therefore, to exclude any effect on texture analysis originating from innate differences due to the type of US machine used, and not from pathologic differences, we decided to repeat the same process to define a radiomics score in a homogeneous subgroup who had undergone US examinations with iU22 only.

**Table 3:** Diagnostic performance of the radiomics score depending on the type of US machines.

	Training data set				Validation data set			
	IU22	E9	HDI5000	P*	lU22	E9	HDI5000	P*
Total (median, IQR)	−2.07 (−2.79, −1.17)	−1.70 (−2.34, −0.62)	−1.54 (−2.49, −0.96)	0.016	−1.95 (−2.72, −1.04)	−1.51 (−2.43, −0.49)	−1.61 (−2.64, −0.73)	0.003
Benign (mean ± SD)	−2.27 ± 1.14	−2.08 ± 1.20	−2.00 ± 0.94		−2.21 ± 1.08	−1.82 ± 1.22	−2.04 ± 1.21	
Malignancy (mean ± SD)	−0.36 ± 1.09	−0.10 ± 0.91	−0.16 ± 0.98		−0.53 ± 1.34	0.11 ± 1.18	−0.33 ± 0.87	
P^†^	<0.001	<0.001	<0.001		<0.001	<0.001	<0.001	
AUC (95% CI)	0.893 (0.843, 0.944)	0.912 (0.864, 0.960)	0.922 (0.834, 1.000)		0.834 (0.770, 0.898)	0.868 (0.800, 0.937)	0.864 (0.736, 0.992)	

P* value compared among US machines, Kruskal-Wallis test.

^†^P value compared between benign and malignant lesions, Student’s t-test.

SD = standard deviation, IQR = interquartile range.

### Subgroup analysis

**Figure 2:**
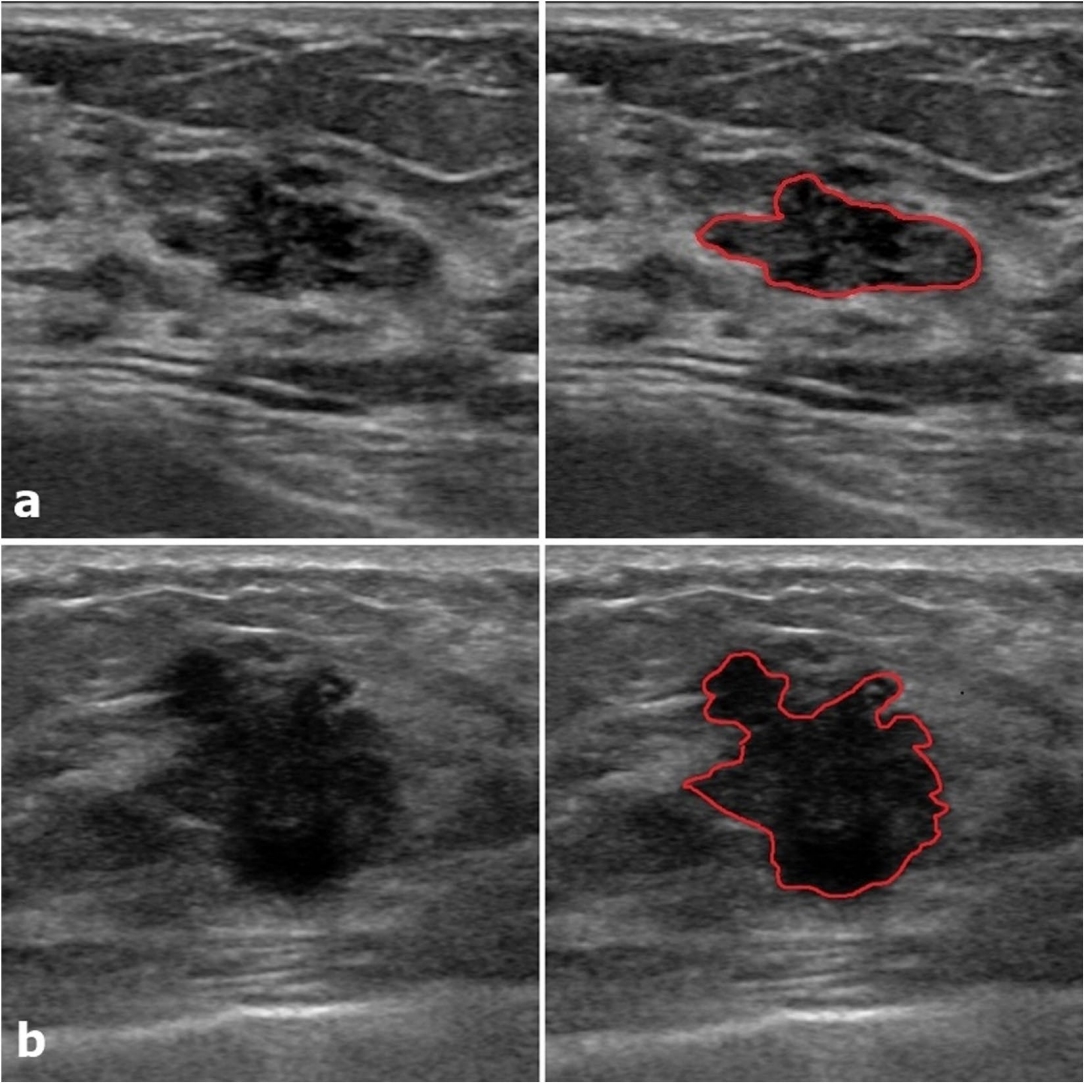
Two representative examples with defined ROI. (**a**) An 18mm-sized oval, microlobulated, hypoechoic lesion (BI-RADS category 4a) which was confirmed as fibroadenoma presented a low radiomics score (−3.83, predicted malignancy 0.4%). (**b**) A 20mm-sized irregular, spiculated, hypoechoic lesion (BI-RADS category 5) which was confirmed as TNBC presented a high radiomics score (3.01, predicted malignancy 99.6%).

**Figure 3:**
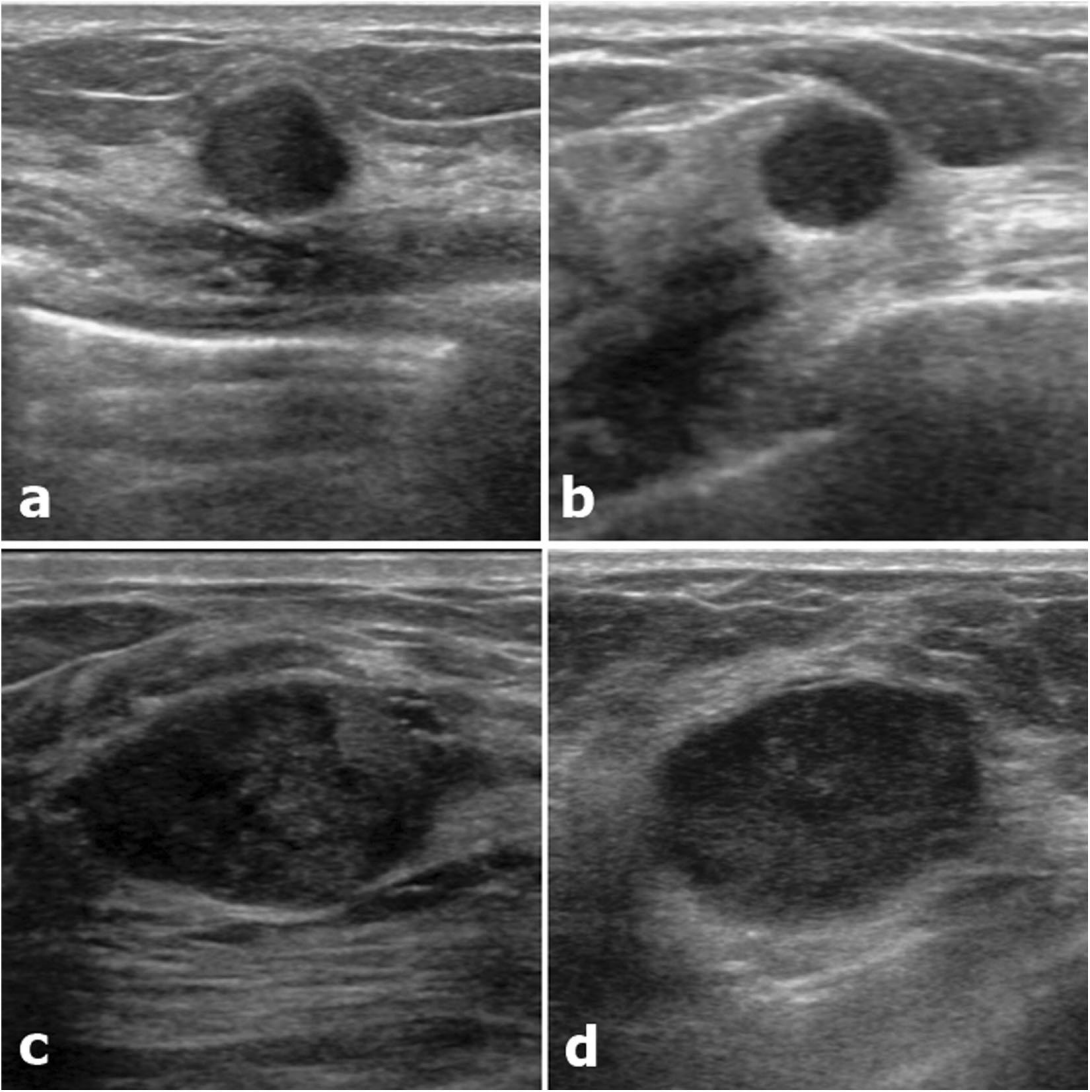
Two couples of similar lesions with differences in the radiomics score. (**a**) A 10mm-sized round, microlobulated, hypoechoic lesion (BI-RADS 4a) confirmed as fibroadenoma showed a radiomics score of −2.19 (predicted malignancy 5%). (**b**) A 9mm-sized round, microlobulated, hypoechoic lesion (BI-RADS 4a) confirmed as TNBC showed a radiomics score of −1.36 (predicted malignancy 17%). (**c**) A 26mm-sized oval, angular, hypoechoic lesion (BI-RADS 4a) confirmed as fibroadenoma showed a radiomics score of −1.11 (predicted malignancy 24%). (**d**) A 25mm-sized oval, microlobulated, hypoechoic lesion (BI-RADS 4a) confirmed as TNBC showed a radiomics score of 0.22 (predicted malignancy 74%).

Twenty-six radiomics features were newly selected by the LASSO analysis in the iU22 subgroup (Supplementary Table S2). Among the 26 features, the feature which suggested TNBC most strongly was LH_srlgle_52_90 (low/high, short run low gray level emphasis, 90 degree) and the feature which favored fibroadenoma most strongly was same as before, LH_imc1_35_90 (low/high, informational measure of correlation 1, 90 degree). The new radiomics score presented a high diagnostic performance for differentiating fibroadenoma and TNBC; AUC (95% CI) 0.910 (0.874, 0.946) in the training set and 0.853 (0.752, 0.954) in the validation set (Fig. 2). Among 415 lesions diagnosed as BI-RADS category 3 or 4a (377 fibroadenomas and 38 TNBCs), in which radiomics scores are expected to aid differential diagnosis, radiomics scores significantly differed according to pathology (*p* < 0.05 in the total, training and validation sets). However, AUC was slightly decreased for these lesions; 0.838 (0.768, 0.907) in the training set and 0.782 (0.599, 0.966) in the validation set (Table 4, Fig. 3).

**Table 4:** Diagnostic performance of the radiomics score in the iU22 subgroup.

	ALL		BI-RADS 3 + 4a		
	Training set	Validation set	Training set	Validation set	Total
Number of lesions	369	123	313	102	415
Benign (median, IQR)	−2.33 (−3.16, −1.60)	−2.36 (−3.23, −1.84)	−2.38 (−3.19, −1.68)	−2.37 (−3.34, −1.86)	−2.37 (−3.22, −1.71)
Malignancy (median, IQR)	−0.22 (−1.14, 0.36)	−0.61 (−1.40, 0.46)	−1.14 (−1.59, −0.44)	−1.35 (−1.74, −0.04)	−1.17 (−1.65, −0.38)
P^†^	<0.001	<0.001	<0.001	0.002	<0.001
AUC (95% CI)	0.910 (0.874, 0.946)	0.838 (0.768, 0.907)	0.853 (0.752, 0.954)	0.782 (0.599, 0.966)	0.821 (0.750, 0.892)

^†^P value compared between benign and malignant lesions, Mann-Whitney U test.

IQR = interquartile range.

## Discussion

TNBC, an aggressive subtype of breast cancer, requires fast and accurate diagnosis. However, in some cases, diagnosis might be delayed due to rather benign morphology on grayscale US^9–13^. We tried to investigate whether a radiomics score based on texture analysis can help differential diagnosis in these types of cases where malignancy is indiscernible with the naked eye. Moon *et al*.^25^ proved that conventional texture features combined with invariant texture features by ranklet transformation presented a very high diagnostic accuracy in discriminating TNBCs from fibroadenomas (AUC 0.970); however, their study was performed with a relatively small sample size using a single US platform. We tried to reproduce this result in a large number of lesions by using a more simple method that excluded the complicated process of segmentation and ranklet transformation, while also taking into consideration the BI-RADS categories.

When we applied a radiomics score defined in a total of 901 lesions with three different US machines, the radiomics score showed significant difference between benign and malignant lesions for each US machine (*p* < 0.001). Most of the selected radiomics features were wavelet-based features which were presumed to redisplay tumor characteristics hidden behind the speckle and show discriminative ability^26^. However, median values differed depending on the type of US machine used, even though there was no considerable difference in the pathologic ratio. This was considered to be originated from innate differences among the US platforms such as brightness or contrast, although we set the dynamic range to be equal. Most previous studies about texture analysis obtained images with a single US machine^18,25,27–29^, and a previous study additionally showed that different sonographic platforms might affect the consistency of diagnostic performance in smaller groups, especially in wavelet transformation^30^. This issue is associated with standardization of data acqusition, which is crucial point in radiomics research regarding the potential lack of reproducibility^31^. It is relatively more attainable in CT or MRI by setting the scan parameters or pulse sequences identically. That is why radiomics studies for breast cancer were also done by mostly using MRI^21^. However, US is much more easily accessible and effective tool for breast tumor screening and diagnosis, and a few previous studies dealt with more robust US features regardless of US platforms or gray-scale variations^30,32,33^. Our study had significance in that it reflected clinical situation using different kinds of US platforms and the result that even this platform-dependent radiomics score presented high diagnostic performance might imply generalizable potency of the radiomics score.

To reduce the effect of the US machine itself on texture analysis, we separately developed a radiomics score in a single major subgroup which was made up of patients examined with one machine, using the same LASSO analysis. This new radiomics score also differentiated between fibroadenoma and TNBC with high diagnostic accuracy (AUC 0.910 in the training set, 0.838 in the validation set; Table 4). However in the clinical field, lesions diagnosed as BI-RADS category 4b, 4c or 5 are morphologically distinct enough for malignancy to be suspected even without the radiomics score. When we obtained the AUC in BI-RADS category 3 and 4a lesions, it was slightly lower than the previous AUC of the total lesions (0.853 in the training set, 0.782 in the validation set; Table 4). In a previous study on category 3 lesions, the AUC was 0.58 by conventional texture analysis and upgraded to 0.83 by ranklet transformation^33^. The latter result was similar to our result. In some cases in which fibroadenoma and TNBC were hardly distinguishable to the naked eye, the radiomics score presented substantial differences in the predicted probability of malignancy (Fig. 3). Subsequent research seems to be required to assess the incremental value of radiomics scores in the clinical field, especially for BI-RADS category 3 and 4a lesions.

There were several limitations to this study. First, retrospective radiomics research based on US texture features cannot completely overcome the operator dependency of the initial examination. Further studies are required to standardize US measurements for more widely accessible and reproducible radiomics score. Second, we manually defined the ROIs for each lesion, which possibly yielded interobserver variablity although the two radiologists in our study showed good interobserver agreement. Recently, differential diagnosis of breast lesions based on deep learning showed higher diagnosic accuracy than conventional CAD, with potential errors due to defining ROIs or different US platforms being avoided^34,35^. Finally, additional reader studies in independent validation set is required to compare diagnostic performance between radimoics score and radiologists and evaluate how these results can be applied in actual clinical diagnosis.

In conclusion, a radiomics score based on US texure analysis presented a high diagnostic performance in the differential diagnosis of fibroadenoma and TNBC, even in BI-RADS category 3 and 4a lesions. It was expected to assist radiologist’s diagnosis and reduce the number of invasive biopsies, although US standardization should be overcome before clinical application.

## Electronic supplementary material

Supplementary Information
