# Supplementary material for: Radiomics of US texture features in differential diagnosis between triple-negative breast cancer and fibroadenoma
Source: Sci Rep. 2018 Sep 10;8:13546. doi: 10.1038/s41598-018-31906-4 (PMC6131410; doi:10.1038/s41598-018-31906-4)
Supplement: Supplementary file 1 — Supplementary Information [file 41598_2018_31906_MOESM1_ESM.docx]

**Radiomics of US texture features in differential diagnosis between triple-negative breast cancer and fibroadenoma**

Si Eun Lee, MD^1^, Kyunghwa Han, PhD^1^, Jin Young Kwak, MD, PhD^1^, Eunjung Lee, PhD^2^ and Eun-Kyung Kim MD, PhD^1^

^1^Department of Radiology, Severance Hospital, Research Institute of Radiological Science and Center for Clinical Image Data Science, Yonsei University College of Medicine, Seoul, Korea

^2^Department of Computational Science and Engineering, Yonsei University, Seoul, Korea

**Supplementary information**

**Supplementary Figure S1.** Tuning parameter (lambda) selection in the LASSO model used 10-fold cross-validation. The area under the receiver operating characteristic (AUC) curve was plotted versus log (lambda). Dotted vertical lines represent standard error. A log (lambda) value of -3.899 was chosen (maximum AUC criteria) according to 10-fold cross-validation. A coefficient profile plot was produced against the log (lambda) sequence. A vertical line was drawn at the selected value using 10-fold cross-validation, which resulted in 23 nonzero coefficients.

**Supplementary Table S2**. Selected 23 coefficients in the total lesions.

**Supplementary Table S3.** Newly selected 26 coefficients in the subgroup analysis (iU22).

**Supplementary Figure S1.**


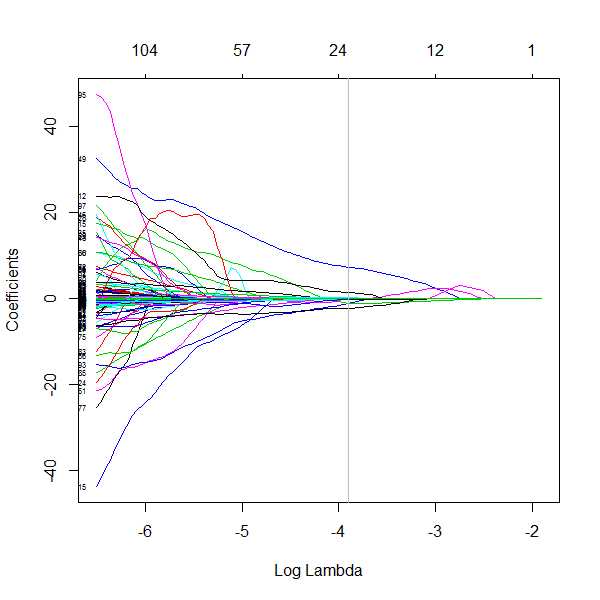

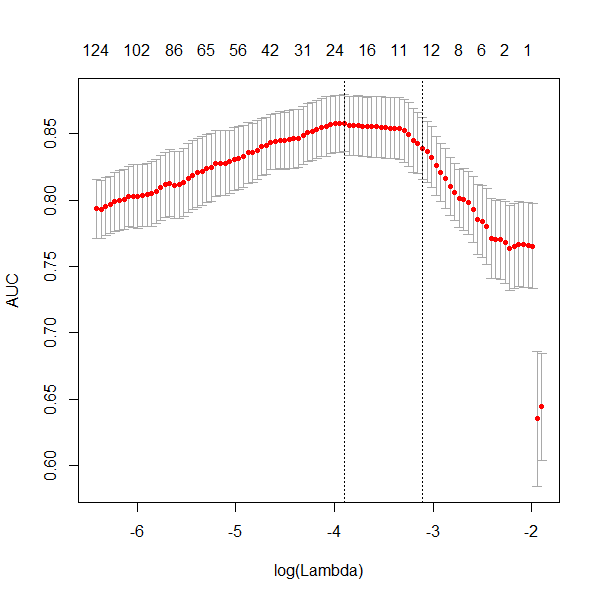


**Supplementary Table S2.**

| **Variables** | **coefficient** | **Variables** | **coefficient** |
| --- | --- | --- | --- |
| (Intercept) | -2.3316300 | LH_lrhgle_55_0 | -0.0002521 |
| mad_6_0 | -0.1374864 | LH_imc1_35_90 | -2.2653466 |
| imc1_35_90 | -1.1566518 | LH_imc1_35_135 | -0.1345481 |
| lre_46_90 | 0.0093246 | LL_mad_6_0 | -0.0071841 |
| HH_de_29_0 | -0.0195407 | LL_med_7_0 | -0.0011124 |
| HH_de_29_45 | -0.3112285 | LL_rms_10_0 | 0.1309122 |
| HH_srhgle_53_45 | -0.0134659 | LL_var_14_0 | -0.0252005 |
| HH_sre_45_90 | 7.3104681 | LL_imc2_36_0 | 1.3534682 |
| LH_kurt_3_0 | 0.0328867 | LL_srhgle_53_0 | -0.0021741 |
| LH_mad_6_0 | -0.0742089 | LL_lrlgle_54_0 | -0.0059057 |
| LH_std_12_0 | -1.0965135 | LL_de_29_45 | -0.0394601 |
| LH_var_14_0 | -0.0000341 | LL_srhgle_53_90 | -0.0220483 |

**Supplementary Table S3.**

| **Variables** | **coefficient** | **Variables** | **coefficient** |
| --- | --- | --- | --- |
| (Intercept) | -7.3812366 | HL_sre_45_135 | 5.8996119 |
| mad_6_0 | -0.1760653 | LH_de_29_45 | 0.7582964 |
| se_42_135 | -0.0669906 | LH_imc1_35_90 | -2.1056074 |
| lre_46_90 | 0.0036042 | LH_se_42_90 | -1.0383084 |
| HH_mean_5_0 | -0.5718675 | LH_sv_43_90 | 0.0013197 |
| HH_sv_43_90 | -0.0011239 | LH_srlgle_52_90 | 13.9030338 |
| HH_lre_46_90 | 0.001475 | LL_rms_10_0 | 0.0277397 |
| HH_imc1_35_135 | 0.5002611 | LL_std_12_0 | -0.1300195 |
| HL_med_7_0 | -0.1877224 | LL_var_14_0 | -0.0228756 |
| HL_lrhgle_55_90 | 0.0089163 | LL_imc1_35_0 | -0.2616723 |
| HL_se_42_135 | -0.5531183 | LL_imc2_36_0 | 7.094894 |
| HL_sv_43_135 | 0.0052489 | LL_lrlgle_54_0 | -0.0077086 |
| LL_de_29_45 | -0.6251203 | LL_sv_43_90 | 0.009126 |
| LL_srhgle_53_90 | -0.0204544 |  |  |
